# Clinical characterization of acute and convalescent illness of confirmed chikungunya cases from Chiapas, S. Mexico: A cross sectional study

**DOI:** 10.1371/journal.pone.0186923

**Published:** 2017-10-24

**Authors:** Rogelio Danis-Lozano, Esteban Eduardo Díaz-González, Karina del Carmen Trujillo-Murillo, Sandra Caballero-Sosa, Jesús Sepúlveda-Delgado, Iliana Rosalía Malo-García, Luis Miguel Canseco-Ávila, Luis Manuel Salgado-Corsantes, Sergio Domínguez-Arrevillaga, Raúl Torres-Zapata, Omar Gómez-Cruz, Ildefonso Fernández-Salas

**Affiliations:** 1 Centro Regional de Investigación en Salud Pública, Instituto Nacional de Salud Pública, Tapachula, Chiapas, México; 2 Facultad de Ciencias Biológicas, Universidad Autónoma de Nuevo León, San Nicolás de los Garza, Nuevo León, México; 3 Centro de Investigación y Desarrollo en Ciencias de la Salud, Universidad Autónoma de Nuevo León, Monterrey, Nuevo León, México; 4 Hospital Regional de Alta Especialidad “Ciudad Salud”, Secretaría de Salud, Tapachula, Chiapas, México; 5 Facultad de Ciencias Químicas, Universidad Autónoma de Chiapas, Tapachula, Chiapas, México; 6 Clínica Hospital “Dr. Roberto Nettel”, Instituto de Seguridad y Servicios Sociales de los Trabajadores del Estado, Tapachula, Chiapas, México; CEA, FRANCE

## Abstract

**Background:**

The emerging chikungunya virus (CHIKV), is an arbovirus causing intense outbreaks in North America. The situation in Mexico is alarming, and CHIKV threatens to spread further throughout North America. Clinical and biological features of CHIKF outbreaks in Mexico have not been well described; thus, we conducted a cross sectional study of a CHIKV outbreak in Chiapas, Southern Mexico to further characterize these features.

**Methodology/Principal findings:**

We collected blood samples from patients suspected of having chikungunya fever (CHIKF) who presented to Clinical Hospital ISSSTE Dr. Roberto Nettel in Tapachula, Chiapas, Mexico. In addition to the clinical examination, real-time polymerase chain reaction (PCR) standardized for the Asian Chikungunya lineage and/or enzyme-linked immunosorbent assay for immunoglobulin M (IgM) were used to confirm CHIKV diagnosis. Of a total of 850 patients who presented with probably CHIKV at Hospital “Dr. Roberto Nettel”, 112 probable CHIKF cases were enrolled in this study from November 2014- June 2015, of which 95 patients (84.8%) were CHIKV positive and 17 were negative (15.2%). Of these 95 CHIKV positive patients, 62 were positive by real-time reverse transcriptase PCR (+qRT-PCR); and 33 were seropositive to +IgM with a negative qRT-PCR. The most frequent symptoms reported were fever (100%), headache (82.3%), polyarthralgia (72.1%), and exanthem (82.3%). Biological abnormalities observed during CHIKV infection were lymphopenia (41.1%), leukopenia (51.6%), elevated transaminases (30.5%-46.3%) and high LDH (46.3%) and CRP (60.0%).

**Conclusion:**

Clinical and biological data obtained from this study is providing more useful information for benchmarking purposes with outbreaks from different parts of the world and would be helpful for better patient care and treatment.

## Introduction

Chikungunya virus (CHIKV) is a febrile threat that has been attacking the New World since 2013. Chikungunya fever (CHIKF) is caused by the chikungunya virus (CHIKV) that belongs to the *Togaviridae* family, *Alphavirus* genus, and is transmitted by the *Aedes aegypti* and *Aedes albopictus* mosquitoes [1]. Since its introduction to the New World, CHIKV has caused impressive outbreaks throughout the American continents, with 1,118,763 cases during the 2013–2014 epidemics [2]. In the following years, CHIKF has caused 575,281 cases in 2015; 998,015 cases in 2016 and 140,550 cases up to epidemiological week 30 in 2017, affecting mainly in Central America and South America [3]. After a year of chikungunya emergence in the Americas, this arbovirus entered Mexico in October 2014 through the Mexico-Guatemala border in Chiapas State [4], but was not reported officially until November of the same year [5]. In Mexico, the Asian genotype of the CHIKV is circulating, and transmission is constrained to *Ae*. *aegypti* [6–8]. The Asian tiger mosquito, *Ae*. *albopictus*, has not been identified as a vector of CHIKV and *Ae*. *albopictus*-adaptive mutations have not appeared yet in Mexico [4,9]. Since its introduction to Mexico, CHIKV has followed three paths of spread emergence from its point of entry, Chiapas State: along the Pacific coast, the Gulf of Mexico coast, and the Yucatan Peninsula. During the 2015 outbreak, through epidemiological week 38, the most affected States were Guerrero (20.4%), Michoacán (15.4%), Oaxaca (14.8%), and Veracruz (11.8%), while the remaining cases (37.6%) were distributed mainly in the states of Chiapas, Colima, Yucatán, Morelos, and Campeche [10].

The clinical manifestations of CHIKV are well described in previous studies and reports of the outbreaks in Africa [11–14], Europe [15], and Asia [16–18]. Of the outbreaks in the Americas, only CHIKF outbreaks in Colombia, Trinidad and Grenada have been described in detail [19–21]. Typically, the onset of CHIKF is abrupt and includes the following symptoms: fever, severe polyarthralgia, headache, back pain, and myalgia. Fever is generally the first symptom to appear; the other symptoms follow within a few days. Arthralgia usually affects more than one joint; and the most affected joints are the knees, ankles, hands, and wrists. The symptoms typically resolve within 7–10 days, although joint pains can last weeks, months, or years. Laboratory features during a CHIKV infection have been described in reports from various outbreaks. The most significant features seen in CHIKV-positive patients are lymphopenia, leukopenia, hypocalcemia, and elevated transaminases; but these features were not consistently reported in all studies and vary depending the clinical phase of infection [12,13,20,22]. Clinical variation could be explained by unique immunopathogenic responses of the two CHIKV genotypes. Evidence of the lesser virulence and pathogenicity of the Caribbean lineage (Asian genotype) compared with the Indian Ocean lineage, which evolved from the Eastern/Central/Southern African (ECSA) genotype, was recently obtained using a mouse infection model [23].

The clinical presentation good outcomes based on genotype/lineage have been poorly discussed. This study is an attempt to document and describe the clinical and biological features of CHIKV outbreak caused by the Caribbean linage in Mexico. In addition, we compared clinical descriptions of outbreaks caused by the ECSA-IOL and Asian genotype worldwide with the results obtained in our study. We conducted a cross-sectional observational study in a secondary healthcare hospital in Tapachula, Chiapas, Mexico, at which probable CHIKV cases were recruited and then diagnosed by molecular and serological testing. Additionally, clinical symptomatology was recorded by each enrolled patient and then blood cell count and biochemical analysis were performed to obtained relevant biological data related to CHIKV infection.

## Methods

### Study area

A cross-sectional and observational study was carried between December 2014 and June 2015 in the Clinical Hospital “Dr. Roberto Nettel Flores” located in the city of Tapachula (14°56’ N, 92°17’ W), Chiapas State, near 15km from the Mexico-Guatemala border. This hospital attends patients form Soconusco region, composed by Tapachula and other 15 municipalities: Metapa, Acapetahua, Acacoyagua, Escuintla, Frontera Hidalgo, Huehuetan, Huixtla, Union Juarez, Mazatan, Cacahoatan, Villa Comatitlan, Suchiate, Tuxtla Chico, Tuzantan and Mapastepec. This region has a population of 728,647, and dengue fever (DENF) has been historically endemic [24,25] in this area.

### Ethical considerations

The study was approved by the Bioethical Committee of the National Institute of Public Health (Project #1312 and approval #1738). Patients were enrolled in the study during a CHIKV outbreak, and blood samples were collected as part of the routine laboratory analysis of patients admitted to Hospital Dr. Roberto Nettel (S1 File). Therefore, only an oral consent to participate in the study was obtained from each patient. All pertinent information about the study (purpose, procedures, risks, benefits, alternatives to participation, etc.) was explained to potential participants in the presence of an independent witness, and every patient who agreed to participate was recorded in an intake form by the hospital’s Epidemiology Department [26].

### Study population and recruitment

The Hospital “Dr. Roberto Nettel” attends only State workers and their immediate relatives who represent 4.5% of the total population of Soconusco Region [27]. Patients who were admitted in this hospital were included in the study if their clinical symptoms met the following inclusion criteria for suspicion of CHIKF: acute onset of fever >38.5°C accompanied by severe arthralgia not explained by other medical conditions in patients residing in the epidemic area [28]. Exclusion criteria were if patients disagree to participate in the study or were DENV-positive by RT-PCR or ELISA.

### Clinical examination and blood collection

After enrollment, a clinical examination was performed following the epidemiological case study format for CHIKV provided by the General Direction of Epidemiology, Ministry of Health [26]. This format collects presence or absence of following symptoms: fever, polyarthralgia, arthritis, headache, myalgia, edema, exanthema, adenopathies, retroorbital pain, digestive alterations (vomiting, diarrhea, and nausea), etcetera. To avoid any confusion between polyarthralgia and arthritis, we differentiated them being the first a transient, intermittent, or persistent pain in multiple joints while the second is the inflammation of one or more joints. Additional epidemiological data such as patient age, gender and travel history is also included. After the clinical examination, the physician filled the format by patient and then collected three blood samples from each patient: one tube with EDTA for a complete blood cell count and one tube with citrate and one dry-tube for the remaining laboratory tests. Dry-tubes were centrifuged at 3,000 rpm, and serum was aliquoted and stored at −80°C for testing.

### Clinical laboratory analysis

Hematological parameters including erythrocytes, hemoglobin, hematocrit, mean corpuscular volume, mean corpuscular hemoglobin, mean corpuscular hemoglobin concentration, white blood cells (WBC), mean platelet volume, and red cell distribution width were determined using a hematology analyzer Advia^®^ 120 (Siemens Healthcare Diagnostics, Erlangen, Germany). Other parameters, including differential WBC (%), prothrombin time (PT), activated partial thromboplastin time (PTT), and fibrinogen, were determined using a coagulation analyzer IL ACL Elite PRO (Diamond^®^ Diagnostics, Holliston, MA). Laboratory assessment of liver function was determined by measuring aspartate aminotransferase (AST), alanine aminotransferase (ALT), alkaline phosphatase (ALP), lactate dehydrogenase (LDH), γ-glutamyltransferase, total protein, albumin, globulin, albumin/globulin ratio, total bilirubin, direct bilirubin, and indirect bilirubin using an Integrated Chemistry System Dimension^®^ RxL Max^®^ (Siemens Healthcare Diagnostics, Erlangen, Germany). Others biochemical parameters, including glucose, urea, creatinine, uric acid, triglycerides, total cholesterol, serum electrolytes (sodium, chloride, and potassium), and inflammatory marker C-reactive protein (CRP) were measured using Dimension^®^ RxL Max^®^.

### Diagnostic qRT-PCR and serology

RNA was extracted from 140μL of serum from each sample using the QIAmp^®^ Viral RNA Mini Kit (Qiagen, Hilden, Germany) according to the manufacturer’s protocol. For molecular diagnostics, one-step qRT-PCR was performed according to the Center of Disease Control (CDC) protocol using primers and probe designed for Caribbean lineage (Asian genotype) [29]: 1) we used the primers CHIK 856 5’-ACCATCGGTGTTCCATCTAAAG-3’ and CHIK 962c 5’-GCCTGGGCTCATCGTTATT-3’ and the TaqMan Probe CHIK 908 5’-FAM- ACAGTGGTTTCGTGTGAGGGCTAC-NFQ-3´; 2) the qRT-PCR was prepared with the QIAGEN QuantiTect Probe RT-PCR kit^®^ (Qiagen, Venlo, Netherlands) in accordance with the manufacturer’s protocol, adding 10μL of RNA sample per reaction in duplicate; and 3) the samples were run in the 7500 Fast Real Time PCR System (Applied Biosystems, Foster City, CA). Thermal cycling conditions were: one cycle of 50°C for 30 minutes and 95°C for 15 minutes followed by 45 cycles of 95°C for 15 seconds and 60°C for 60 seconds. The cycle threshold (CT) value was 38: results were considered positive if less than 38 cycles were needed in both wells; negative if greater than 38 cycles were needed in both wells, and equivocal if less than 38 cycles were needed in one of the two wells. Serum samples were screened for anti-CHIKV IgM antibodies by enzyme-linked immunosorbent assay (ELISA) as previously described [30,31] using the CHIKjj Detect MAC-ELISA kit (InBios, Inc., Seattle, WA). All samples were tested in duplicate and any inconclusive samples were retested.

For dengue diagnosis, the samples were separately sent to the State Laboratory of Public Health of Chiapas. Serum was tested with Panbio^®^ Dengue Early ELISA and Panbio^®^ Dengue IgM Capture ELISA (Alere, Waltham, MA) for detection of NS1 antigen and IgM antibody following the manufacturer’s protocol.

## Results

**Fig 1:**
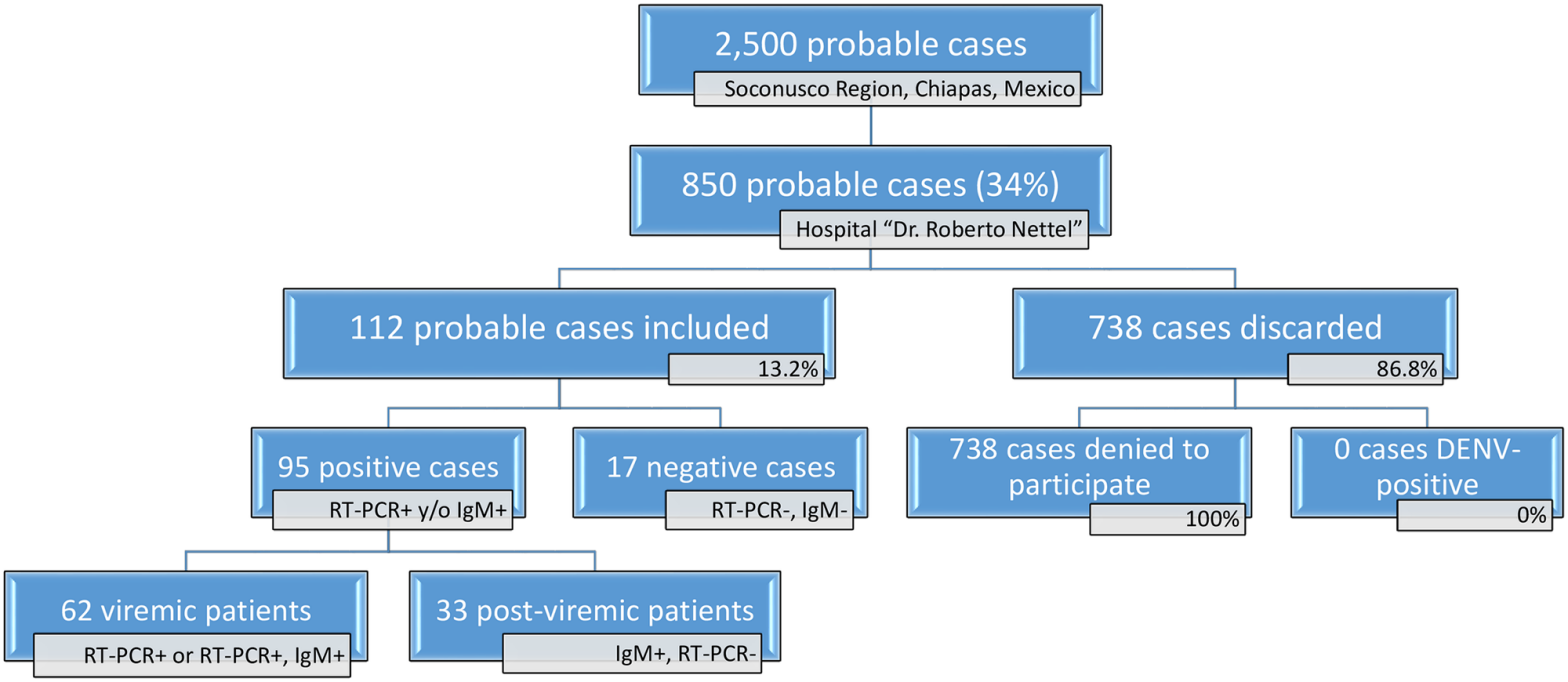
Patient enrollment in Hospital Dr. Roberto Nettel for clinical and laboratory evaluation of the 95 confirmed CHIKF cases. Abbreviation: CHIKF, chikungunya fever.

Ministry of Health reported 2,500 probable chikungunya cases distributed in Soconusco Region from November 2014 to June 2015, of which 850 were admitted by Hospital “Dr. Roberto Nettel”. Of these patients, only 112 agreed to enrollment in this study (Fig 1). Complete clinical, epidemiological, and biochemical data were obtained from each participant; and none was excluded because of missing data. Ninety-five patients had confirmed CHIKF with 62 being +qRT-PCR and 33 +IgM/-qRT-PCR, while the remaining 17 patients were CHIKV-negative (Fig 1). Only one patient was qRT-PCR and IgM positive and was categorized as a viremic case in subsequent data analysis. None of the confirmed CHIKV cases was DENV-positive by NS1 or IgM ELISA. Confirmed CHIKV cases from this study were distributed across nine municipalities, with the greatest number of cases in Tapachula (39 cases, 41.0%), Tuxtla Chico (17 cases, 17.9%), Huehuetan (17 cases, 17.9%), and Cacahoatan (11 cases, 11.6%). Data are shown only for viremic and post-viremic cases because the numbers of negative cases were too few (17 cases) for meaningful analysis.

Demographic data for the 95 patients with confirmed CHIKV are shown in Table 1. The mean age of patients was similar at 37.2 and 39.2 years for viremic and post-viremic patients, respectively. The age groups most affected by CHIKV infection were adults 31–40 and 41–50 years, together accounting for almost half of the CHIKV cases in both phases of disease; however, minors (<20 years) accounted for 12.6% of confirmed cases in both stages. Furthermore, sex distribution was unequal showing a male/female rate of 0.4. Approximately 70% of patients in both phases of disease were female (Table 1).

**Table 1:** Demographic data from the 95 enrolled patients.

	Viremic (+qRT-PCR)		Post-viremic (+IgM)		Total	
Data population	Mean	95% CI	Mean	95% CI	Media	95% CI
Age	37.2	± 3.7	39.2	± 5.1	37.9	±3.0
**Age groups**	N	%	N	%	N	%
0–20 y	8	12.9	4	12.1	12	12.6
21–30 y	10	16.1	5	15.2	15	15.8
31–40 y	13	21.0	8	24.2	21	22.1
41–50 y	18	29.0	9	27.3	27	28.4
>50 y	13	21.0	7	21.2	20	21.1
**Sex**	N	%	N	%	N	%
Female	44	71.0	24	72.7	68	71.6
Male	18	29.0	9	27.3	27	28.4

Abbreviations: qRT-PCR, real-time reverse transcriptase polymerase chain reaction; IgM, immunoglobulin M; CI, confidence interval.

An extensive clinical description of the patients during the viremic and post-viremic phases is described in Table 2. The timing of clinical data and blood collection was an important issue considered in the study. The median numbers of days between symptom onset and consultation (SOC) were 3.0 and 4.0 for viremic and post-viremic patients, respectively. Despite this short time difference in SOC between both phases, we observed some interesting differences in clinical and laboratory features between the disease phases. The most common symptoms recorded during the viremic stage were fever (100%), polyarthralgia (100.0%), headache (82.3%), exanthem (82.3%), chills (82.3%), and nausea (71.0%). Other symptoms often observed in the viremic patients included pruritus (66.1%), arthritis (58.1%), abdominal pain (46.8%), edema (43.5%) and diarrhea (33.9%). On the other hand, the most frequent symptoms described in post-viremic patients were polyarthalgia (100%), headache (87.9%), exanthema (81.8%), chills (75.8%), and nausea (48.5%) and other symptoms developed, such as arthritis (78.8%) and adenopathy (60.6%). The only symptoms that showed substantial differences between phases were arthritis, adenopathy, and nausea (Table 2).

**Table 2:** Clinical features of the 95 confirmed CHIKV cases separated by phase of infection, viremic or post-viremic.

	Viremic (+qRT-PCR)		Post-viremic (+IgM)		Total	
	Median	95% CI	Median	95% CI	Median	95% CI
Days between onset of symptoms and consultation	3.0	2.0–3.0	4.0	2.0–4.0	3.0	2.0–3.0
**Clinical features**	N	%	N	%	N	%
Fever	62	100	33	100	95	100
Headache	51	82.3	29	87.9	80	84.2
Myalgia	4	6.5	0	0.0	4	4.2
Polyarthralgia	62	100	33	100	95	100
Arthritis	36	58.1	26	78.8	62	65.3
Retroorbital pain	9	14.5	3	9.1	12	12.6
Exanthem	51	82.3	27	81.8	78	82.1
Pruritus	41	66.1	26	78.8	67	70.5
Vomiting	11	17.7	1	3.0	12	12.6
Nausea	44	71.0	16	48.5	60	63.2
Chills	51	82.3	25	75.8	76	80.0
Photophobia	1	1.6	0	0.0	1	1.1
Abdominal pain	29	46.8	12	36.4	41	43.2
Diarrhea	21	33.9	14	42.4	35	36.8
Conjunctivitis	10	16.1	5	15.2	15	15.8
Nasal congestion	9	14.5	9	27.3	18	18.9
Cough	9	14.5	4	12.1	13	13.7
Pharyngitis	11	17.7	5	15.2	16	16.8
Splenomegaly	1	1.6	0	0.0	1	1.1
Altered taste	49	79.0	23	69.7	72	75.8
Adenopathy	21	33.9	20	60.6	41	43.2
Eyelid inflammation	4	6.5	1	3.0	5	5.3
Dyspnea	4	6.5	1	3.0	5	5.3
Cardiac alteration	1	1.6	0	0.0	1	1.1
Muscle weakness	15	24.2	5	15.2	20	21.1
Petechiae	0	0.0	1	3.0	1	1.1
Edema	27	43.5	17	51.5	44	46.3
Bleeding	3	4.8	2	6.1	5	5.3

Abbreviations: CHIKV, chikungunya virus; CHIKF, chikungunya fever; qRT-PCR, real-time reverse transcriptase polymerase chain reaction; IgM, immunoglobulin M; CI, confidence interval.

**Fig 2:**
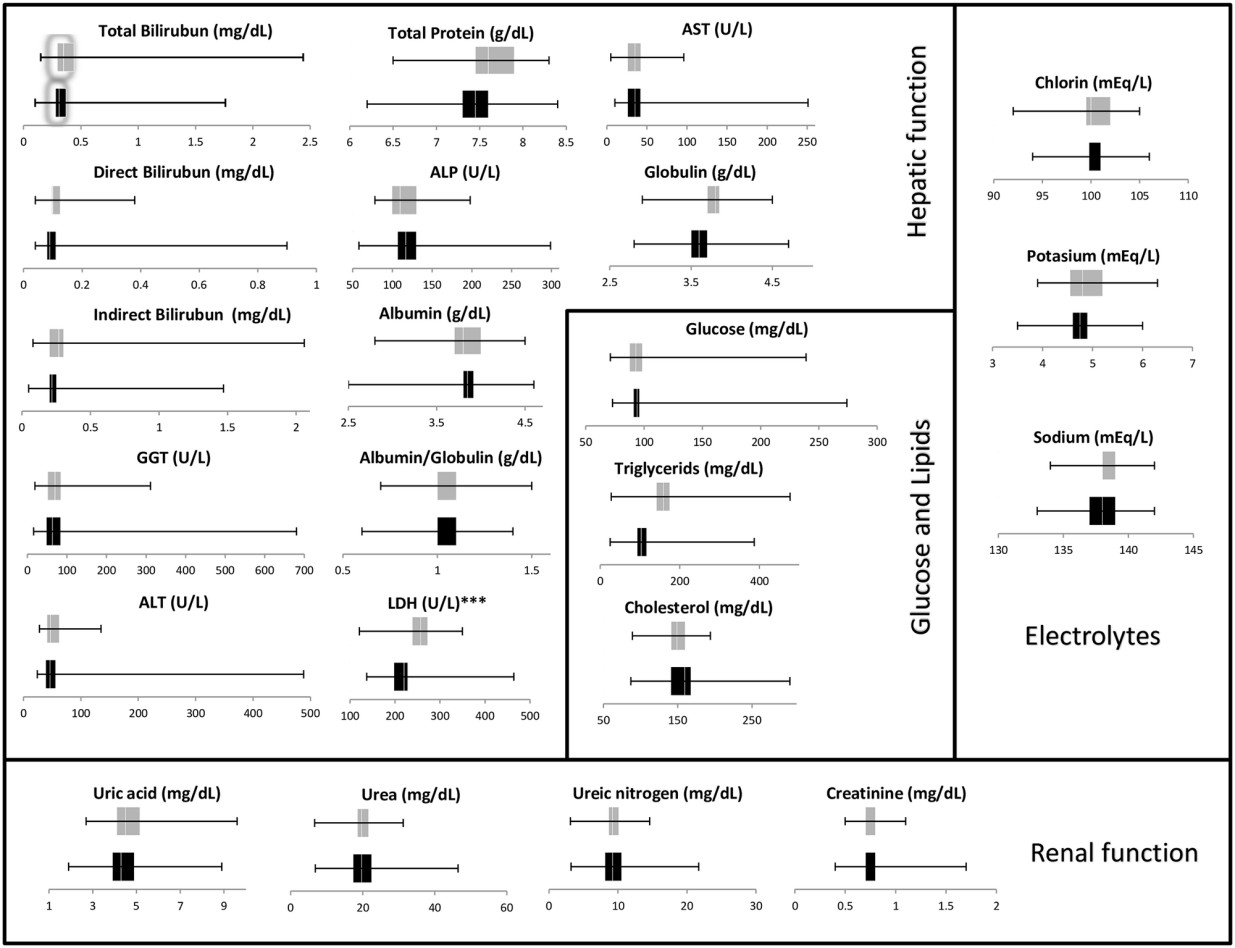
Laboratory values for hepatic and renal function, glucose, lipids, and electrolytes from the 95 confirmed CHIKF cases enrolled in this study. Black bars indicate viremic patients, while grey bars represent post-viremic patients.

**Fig 3:**
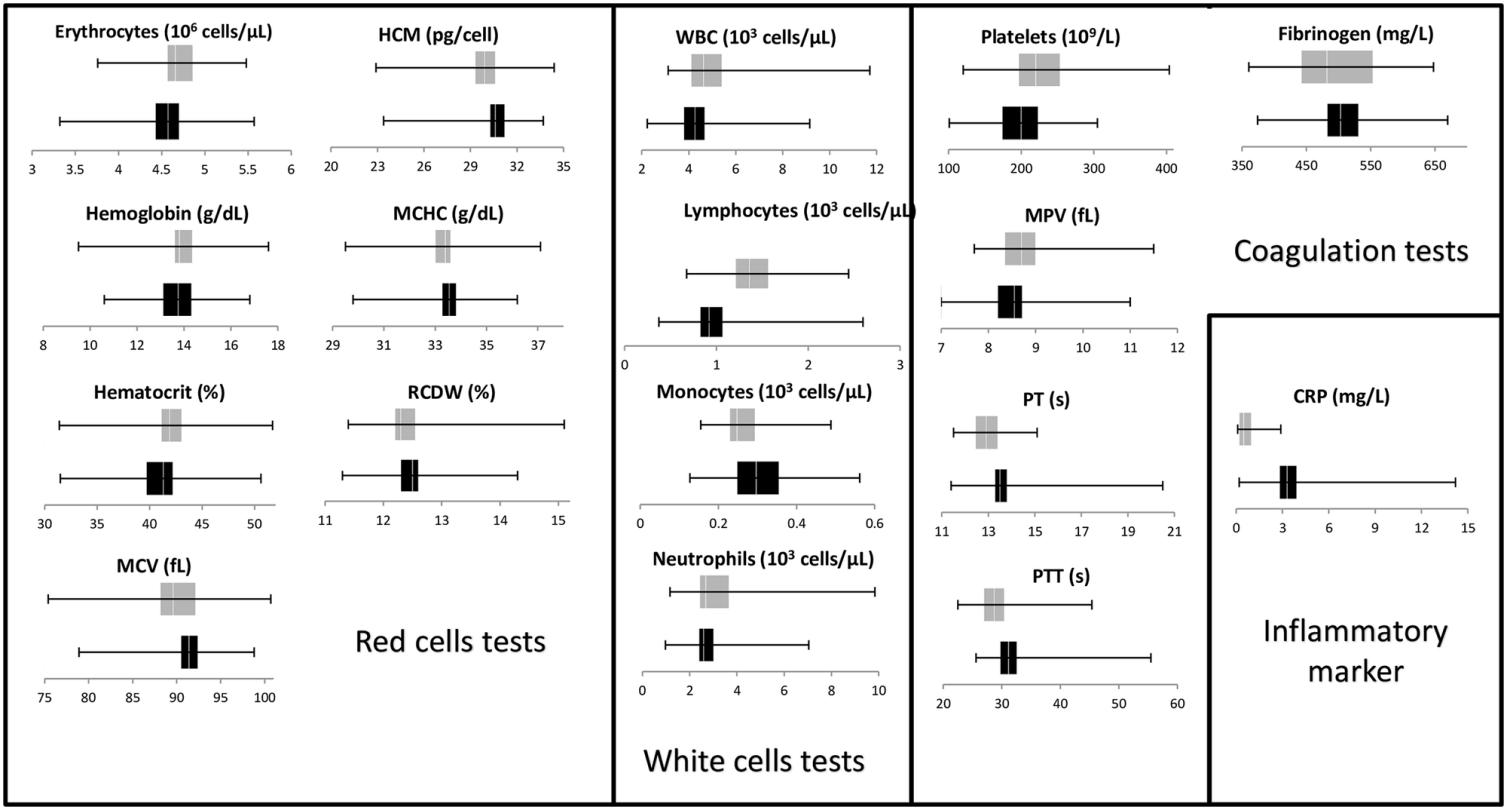
Laboratory values for blood cell counts, coagulation tests, and an inflammatory marker from the 95 confirmed CHIKF cases enrolled in this study. Black bars indicate viremic patients, while grey bars represent post-viremic patients.

Regarding laboratory studies, serum samples were tested for different features: blood cell count, carbohydrates, lipids, electrolytes, inflammatory marker, and hepatic and renal functions. These tests were performed to find any alteration caused by CHIKV infection in the viremic and post-viremic patients, and the results are shown in Figs 2 and 3. Most parameters measured in this study fell within normal ranges in both phases except for hepatic enzymes (AST, ALT, ALP), absolute count of white cells, lymphocytes, LDH and CRP. Of these features, however, notable differences between the two phases were observed in lymphocyte count, LDH and CRP levels. Although differences between the phases were also observed in triglycerides, PT, and PTT, these results were not relevant because most of the values were within normal ranges. Table 3 demonstrates the relevant abnormalities in laboratory values with the respective reference values included. Elevated concentration of hepatic enzymes (AST, ALT, ALP) was seen in approximately 30.6–48.4% and 24.4–42.4% of viremic and post-viremic patients, respectively. Low white cell counts occurred in 60–70% of cases in both disease stages, and lymphopenia was significantly more frequent in viremic patients (56.6%) than in post-viremic patients (12.1%). Moreover, LDH levels were significantly more frequent in post-viremic patients (69.7%) than in viremic patients (33.9%). CRP was expectedly elevated in the viremic patients of infection (61.0%) but normal in all post-viremic patients.

**Table 3:** Biochemical, electrolytic, and hematologic abnormalities observed in the 95 confirmed CHIKV cases.

	Viremic		Post-viremic		Total	
	N	%	N	%	N	%
High AST (>37 U/L)	30	48.4	14	42.4	44	46.3
High ALT (>65 U/L)	19	30.6	11	33.3	30	31.6
High ALP (>136 U/L)	21	33.9	8	24.2	29	30.5
High GGT (>85 U/L)	23	37.1	10	30.3	33	34.7
High LDH	21	33.9	23	69.7	44	46.3
Low A/G (<1.2)	44	71.0	27	81.8	71	74.7
Leukopenia (<4500 cells/mm^3^)	34	54.8	15	45.5	49	51.6
Lymphopenia (<1000 cells/mm^3^)	35	56.5	4	12.1	39	41.1
Monocytopenia (<200 cells/mm^3^)	13	21.0	4	12.1	17	17.9
Thrombocytopenia (<100 x 10^3^ platelets/mm^3^)	11	17.7	5	15.2	16	16.8
Low Hematocrit (<40.0%)	25	40.3	8	24.2	33	34.7
High CRP (>3 mg/dL)	38	61.3	0	0%	38	40.0
Hyponatremia (<136 mEq/L)	9	14.5	5	15.2	14	14.7
Hyperkalemia (>5.1 mEq/L)	10	16.1	12	36.4	22	23.2
Hypochloremia (<98 mEq/L)	9	14.5	4	12.1	13	13.7

Abbreviations: CHIKV, chikungunya virus; CHIKF, chikungunya fever; AST, aspartate aminotransferase; ALT, alanine aminotransferase; ALP, alkaline phosphatase; GGT, γ-glutamyltransferase; LDH, lactate dehydrogenase; A/G, albumin/globulin ratio; CRP, C reactive protein.

## Discussion

At the end of 2014, chikungunya virus emerged in Mexico and, since then, have caused outbreaks in the states along the Pacific and Gulf of Mexico coasts. Previous sequencing data suggests that the CHIKV entered the country through the Mexico-Guatemala border in Chiapas [4]. Precisely in this point of entry, we conducted clinical and laboratory evaluation of 112 patients with symptoms concerning for CHIKF, confirming 95 cases by qRT-PCR and ELISA. Depending on whether the patient was qRT-PCR or IgM positive, the patient was placed into one of two groups, the viremic patients group or the post-viremic patients group. From these patients, we analyzed data from the clinical data and laboratory tests; described basic demographic information, such as age and gender.

The key results were as follows: 1) of 112 probable CHIKF cases, 95 cases (84.8%) were confirmed by qRT-PCR (33) or IgM ELISA (62) being adults and females more affected; 2) fever, polyarthralgia, exanthem, and headache were the most common symptoms observed in patients, however, adenopathies that had not been deeply described in previous studies were also found in our study, 3) in the disease onset, symptomatology between previous IOL outbreaks and Mexican outbreak caused by Asian lineage seems to be similar and 4) biological abnormalities observed during CHIKV infection were lymphopenia, leukopenia, elevated transaminases and high LDH and CRP.

In comparing our results to previously report epidemiological data from other outbreaks, six of nine studies found that the mean or median of age of infected patients was between 30–40 years, which was similar to the mean age of 37.9 years in our study. Most infected patients in most other studies were adults between 20–40 years with percentages between 30 to 50%, with lower frequencies in the elderly and children such as our study. However, two outbreaks from La Reunion Island reported that elderly people between 50–60 years were most affected. Lack of herd immunity in the region may explain why CHIKV infections occurred more often in adults than in old and young patients [32].

The male-to-female ratio in confirmed CHIKV cases has varied. While Thiberville et al. (2009), Reller et al. (2013), and Win et al. (2010) found a higher proportion of affected males, Mattar et al. (2015), Sahadeo et al. (2015), Macpherson et al. (2016) and our study found a higher proportion of infected females. Biased sampling may explain differences among these studies: i.e., Win et al. (2010) selected more immigrant male workers; and Thiberville et al. (2009), Matter et al. (2015), and Sahadeo et al. (2015) each had low numbers of study participants, suggesting that their results are not representative of the general population. The sample obtained for this study is also non-representative and male-to-female ratio of total of probable cases (850 cases) was 0.5 while the ratio of enrolled participants was 0.4 (112 cases). However, it is important to consider that CHIKV transmission might have occurred more in home-environments putting housewives at major risk of infection, such as was described in previous reports of dengue seroprevalence in Mexico [33].

The most common symptoms reported by all previous studies including ours were fever (80–100%), polyarthralgia (80–100%), headache (50–80%), rash (30–70%), and digestive alterations (20–80%). Adenopathy, arthritis, and edema were rarely described in these studies. It is important to point out that exanthem was very high (82.3%) in our study compared with the rest of studies that is seen up to 50% of patients [34].

Cervical lymphadenopathies were observed in Indian CHIKV outbreaks and imported Spanish cases as well as in patients affected by the O’nyong-nyong virus, a related alphavirus [35–37]. Interestingly, Staikowsky et al. (2009) observed an increase of adenopathies from 8.9% in the viremic patients to 29.0% in the post-viremic patients of CHIKV, similar to our study from 33.3% in the viremic phase to 60.6% in the post-viremic phase. The occurrence of lymphadenopathy possibly may have been underdiagnosed in the rest of studies, given that it may occur toward the end of the febrile period, during recovery from the infection, and appears to be self-limiting [37]. Unfortunately, the specific location of adenopathies has not been not recorded in most of studies and neither have we in this study. Regarding arthritis and edema, the lack of sufficient data makes determining the prevalence of these symptoms difficult.

Data including viral genotype from the La Reunion Island, Singapore, Gabon, Sri Lanka, Colombia, and Trinidad & Tobago outbreaks are included in Table 4. Although the Caribbean Asian lineage of CHIKV has been reported as less virulent than the IOL lineage [23], the Asian lineage outbreak from Chiapas and the rest of the ECSA-diverged IOL outbreaks worldwide appear to behave clinically similarly during the first 3–4 days of infection.

**Table 4:** Compilation of the most relevant clinical and abnormal laboratory features during different CHIKV outbreaks worldwide.

Publication	Borgherini et al. 2007	Staikowsky et al. 2009*	Thiberville et al. 2013	Reller et al. 2013	Win et al. 2010	Nkoghe et al. 2012	Mattar et al. 2015	Sahadeo et al. 2015	Macpherson et al. 2016
Location and date	La Reunion (2005–2006)	La Reunion (2005–2006)	La Reunion (2006)	Sri Lanka (2007)	Singapore (2008)	Gabon (2010)	Colombia (2014)	Trinidad & Tobago (2014)	Grenada (2014)
Total number of patients included	157	274	76	797	97	270	100	158	493
CHIKV-positive patients (%)	100	65.7 / 12.4	71.1	3.5	100	100	25.0	22.0	86.0
Genotype / Lineage	ECSA (IOL)	ECSA (IOL)	ECSA (IOL)	ECSA (IOL)	Unknown	ECSA	Asian (Caribbean)	Asian (Caribbean)	Possibly Asian
Age (Mean or Median)	57.9	55.0	40.0	41	37.0	30.0	39	32	34.5
Male / Female proportion	1.2	1.1 / 0.6	1.7	6.8	3.4	0.9	0.6	0.6	0.5
Days post- symptom onset	2.1	1.8 / 6.2	1.2	3.0	2–4	2.0	2.9	2.4	-
Fever (%)	89.0	19.4 / 0.0	100	100	89.7	85.0	100	100	88.6
Arthralgia (%)	96.0	98.8 / 96.8	100	71.0	87.6	90.4	88	83.3	90.0
Myalgia (%)	-	59.0 / 59.0	74.0	82.0	-	72.9	28	70.0	66.7
Headache (%)	47.1	70.0 / 58.8	72.0	75.0	-	71.8	64	76.7	53.9
Rash (%)	40.1	47.8 / 67.7	30–50	11.0	-	41.8	56	33.0	42.9
Digestive alterations (%)^¥^	47.1	63.3 / 85.3	13–44	11.0–38.0	-	32.0	4–6	16–26	26.9
Conjunctivitis (%)	-	22.8 / 17.7	-	36.0	-	-	-	-	-
Adenopathy (%)	8.9	8.9 / 29.4	-	18.0	-	-	8	-	28.8
Bleeding (%)	6.4	1.1 / 8.8	8.0	-	1.0	2.2	-	10.0	-
Arthritis	31.8	-	-	0.0	-	-	-	-	-
Edema	-	25.6 / 44.0	-	-	-	-	-	-	25.1
High Creatinine (>120 μmol/L)^£^	27.4%	111.1±73.1 / 104.2±1099	-	-	-	96.0±32.0	-	-	-
High AST (>37 U/L)^£^	45.9%	55.0±164 / 53.2±42.3	28%	-	3.0%	45.0±35	-	-	-
High ALT (>65 U/L)^£^	16.6%	35.2±89.7 / 39.5±29.9	14.0%	-	8.0%	33.0±17.0	-	-	-
High CRP (>3 mg/dL)^£^	67.5%	5.6±5.0 / 3.1±5.0	33.0%	-	-	-	-	-	-
Leukopenia (<4500 cells/mm^3^)^£^	5153±2198	5431±2139 / 5199±2554	-	4500	3500±1900	-	-	6500±3250	-
Lymphopenia (<1000 cells/mm^3^)^£^	79%	608 ±314 / 1090±481	79.6%	1305	-	2228±216	-	-	-
Thrombocytopenia (<100 x 10^3^ platelets/mm^3^)^£^	9.5%	174.2±56.0 / 173.7±62.2	24.0%	181.0	1.0%	233.1±81.8	-	234.0±72.5	-

*This study reported values for viremic / post-viremic patientss.

^£^ Values may be represented as percentages or means / medians with their respective units depending on the measured parameter.

^¥^ Digestive alterations include abdominal pain, nausea, vomiting, and diarrhea.

Abbreviations: CHIKV, chikungunya virus; ECSA, Eastern/Central/Southern Africa; IOL, Indian Ocean lineage; AST, aspartate aminotransferase; ALT, alanine aminotransferase; CRP, C reactive protein.

Hepatic function was evaluated in this study by measuring the levels of AST, ALT, and ALP. Studies of the outbreaks in La Reunion Island and Gabon reported elevations of these enzymes in approximately 20–46% of cases with AST levels up to 55 and 53.2 U/L in viremic and post-viremic patients, respectively. Our study observed elevations of these enzymes in approximately 30–50% and 24–43% of viremic and post-viremic cases, respectively. Our results are quite similar to those previously reported in the literature, but this increase may be explained by the use of paracetamol and/or ibuprofen for the treatment of symptoms, producing transient drug toxicity in the liver [11]. Another biological feature that has been associated with CHIKV infection is elevated C-reactive protein. Previous studies from the outbreak in La Reunion Island reported high levels of CRP in approximately 50–70% of CHIKF cases, with serum levels up to 5.6 mg/L, similar to the elevated levels of CRP we found in 61.3% of patients disease. Additionally, another enzyme observed raised was LDH in post-viremic patients. This was previously observed elsewhere [38,39] and explained because this enzyme is released into the extracellular environment during cellular injury associated with inflammation [40].

The blood cell count parameters that were abnormally decreased during CHIKV infection were absolute counts of leukocytes and lymphocytes, which occurred in 54.8% and 56.5% of cases, respectively. In post-viremic cases, leukopenia persisted in 45.5% while lymphopenia decreased to 12.1% in the post-viremic patients. Only lymphopenia was reported in 70–80% of confirmed cases in the outbreaks of La Reunion Island, with counts of 608 cells/mm^3^ during the viremic phase. Leukocytosis had been associated with CHIKV infections in the past [41,42]; however, none of the studies cited here found a significant increase in white cells. Moreover, a significant decrease of these cells was observed in Win et al. (2010) and our study.

Limitations of our study must be considered. We obtained a biased sampling, including only patients for one hospital because of the ease of obtaining samples from its clinics, thus, not allowing for a sample that was truly representative of the general population. Another source of bias may be the enrollment of participants. The male/female ratio differed between all patients recorded in the hospital and enrolled participant. Additionally, the age means between all and recruited patients have also relevant difference between both sex, being in males 36.2 and 42.7 years, respectively, while in females were 38.4 and 36.0 years, respectively. Most of previous outbreaks have also faced similar limitations with sample collection, but they were able to undertake meaningful evaluation of the clinical information such as we were able too.

In conclusion, we describe the epidemiology and clinical features of CHIKV infection in Southern Mexico. Clinical data of different outbreaks worldwide would be helpful for the investigation of potential biomarkers for each of the phases of chikungunya fever and therefore, clinical diagnosis and treatment would be improved in viremic and immediate post-viremic phase.

## Supporting information

S1 File Explanation of patient attention during the CHIKV outbreak in the hospital “Dr. Roberto Nettel” of 2014–2015. This appendix clarifies how patients were asked for an oral consent to provide blood samples keeping confidentiality of them as part of routine process of epidemiological surveillance of febrile diseases outbreaks.
(PDF)
