# Supplementary material for: Clinical characterization of acute and convalescent illness of confirmed chikungunya cases from Chiapas, S. Mexico: A cross sectional study
Source: PLoS One. 2017 Oct 24;12(10):e0186923. doi: 10.1371/journal.pone.0186923 (PMC5655440; doi:10.1371/journal.pone.0186923)
Supplement: S1 File — This appendix clarifies how patients were asked for an oral consent to provide blood samples keeping confidentiality of them as part of routine process of epidemiological surveillance of febrile diseases outbreaks. (PDF) [file pone.0186923.s001.pdf]

ISSSTE

Instituto de Seguridad y Servicios Sociales de los Trabajadores del Estado

"2017 AÑO DEL CENTENARIO DE LA PROMULGACION DE LA CONSTITUCIÓN POLITICA DE LOS ESTADOS UNIDOS MEXICANOS"

Editor-in-Chief
Joerg Heber
Public Library of Science: Cambridge, United Kingdom
PLoS ONE

And Academic Editors

Dear Sirs:

The present document has the purpose to give information concerning the samples that were taken to diagnose Chikungunya fever in the study. The Hospital "Dr. Roberto Nettel" belongs to ISSSTE (Instituto de Seguridad Social al Servicio de los Trabajadores del Estado), a healthcare system for government employees in Mexico. ISSSTE hospitals maintain a medical record for all the affiliates in every city they cover. The hospital "Dr. Roberto Nettel" has a permanent program of epidemiological surveillance for exanthemas and febrile diseases to detect possible outbreaks. Every suspected patient attending for a diagnosis of these diseases is asked for a blood sample and, besides the diagnostic procedure, the sample is subjected to other clinical test to maintain periodical medical and clinical records of every registered person in the system. As affiliates to the system, patients are not asked for an informed consent to these actions, since only their assigned hospital will have access to that information to provide the primary care needed. The usual procedure for epidemiological surveillance of exanthemas and febrile disease outbreaks was followed with patients affected by the chikungunya outbreak and attended the hospital in 2014. All patients were confidentially informed about the results of their tests and if other health problems arose from these studies, e.g. elevated glucose or lipids, they were also informed and attended for those health issues.

We hope this explanation clarifies the situation of patient's data reported in the study.

Sincerely

Sandra Caballero, MD.
Epidemiologist
Hospital "Dr. Roberto Nettel", Tapachula, Mexico

I.S.S.S.T.E.
C.H. DR. ROBERTO NETTEL FLORES
TAPACHULA, CHIAPAS.
ISSSTE
04 MAY 2017
